# The psychometric properties and measurement invariance of the Burnout Assessment Tool (BAT-23) in South Africa

**DOI:** 10.1186/s12889-022-13978-0

**Published:** 2022-08-16

**Authors:** Leon T. De Beer, Wilmar B. Schaufeli, Hans De Witte

**Affiliations:** 1 WorkWell Research Unit, Potchefstroom Campus, North-West University, Potchefstroom, 2531 South Africa; 2 Department of Psychology, Utrecht University, 3584 CS Utrecht, The Netherlands; 3 Research Unit Occupational & Organizational Psychology and Professional Learning KU Leuven, 3000 Louvain, Belgium; 4 Optentia Research Unit, Vanderbijlpark Campus, North-West University, Vanderbijlpark, 1900 South Africa

**Keywords:** Burnout, Burnout assessment tool, Latent variable modelling, Measurement invariance

## Abstract

**Background:**

Burnout is an increasing public health concern that afflicts employees globally. The measurement of burnout is not without criticism, specifically in the context of its operational definition as a syndrome, also recently designated as such by the World Health Organisation. The Burnout Assessment Tool (BAT-23) is a new measure for burnout that addresses many of the criticisms surrounding burnout scales. The aim of this study is to determine the validity, reliability, and measurement invariance of the BAT-23 in South Africa.

**Method:**

A quantitative, cross-sectional survey, approach was taken (*n* = 1048). Latent variable modelling was implemented to investigate the construct-relevant multidimensionality that is present in the BAT. For measurement invariance, the configural, metric, scalar, and strict models were tested.

**Results:**

The analyses showed that the hierarchical operationalisation of BAT-assessed burnout was the most appropriate model for the data. Specifically, a bifactor ESEM solution. Composite reliability estimates were all well above the cut-off criteria for both the global burnout factor and the specific factors. The measurement invariance tests showed that gender achieved not only strong invariance, but also strict invariance. However, ethnicity initially only showed strong invariance, but a test of partial strict invariance did show that the mean scores could be fairly compared between the groups when releasing certain constraints.

**Conclusions:**

The BAT-23 is a valid and reliable measure to investigate burnout within the Southern African context.

## Introduction

The case for burnout has been well established over the last few decades [1, 2] and the term is now well-known and used in general conversation. The World Health Organization, in the ICD-11 (2022), has classified burnout as an occupational phenomenon, specifically describing burnout as a “…syndrome conceptualized as resulting from chronic workplace stress that has not been successfully managed” [3]. Most people have also encountered someone who has been said to be affected adversely by work-related factors described as being ‘burned out’. Indeed, burnout has been linked with various concerning individual outcomes, such as: atherosclerosis [4], depression [5], diabetes [6], and hypertension [7], also in South Africa [8]. Additionally, burnout has also been shown to be negatively related to outcomes important to organisations, such as turnover intention, absenteeism, and productivity [1].

Therefore, the evidence indicates that burnout is an increasing public health concern as it not only incurs individual costs but translates into a burden for healthcare systems (both public and private), impeding organisational success and by extension a country’s economic growth. For example, within the South African context, empirical evidence has been presented that employees who score high on burnout claimed approximately double the amount from medical insurance compared to those who scored low on burnout [9]. Furthermore, with the severe impact of COVID-19 and accompanying lockdown strategies in most countries, financial efficiencies for organisations and governments will become even more important which can only be achieved with motivated and well-functioning role-players in the economy. To offset hurdles to achieve this, the accurate identification of burnout risks, for intervention, becomes increasingly important.

Without question, the Maslach Burnout Inventory (MBI) has served the world well and is the *go-to* instrument for research and also the identification of burnout – described frequently as the “gold standard” [2]. The WHO also seemingly describes burnout’s components in line with the MBI, that is: exhaustion, cynicism and professional (in)efficacy [3]. But, as research has developed over time some glaring theoretical and practical concerns have been raised: Firstly, the MBI was not initially intended as a tool to help in *diagnosing* burnout, but for use as a research instrument. Secondly, the conceptualisation of burnout has not been universally accepted and different factor structures for the concept’s operationalization have been argued and established ranging from unidimensional, two factor, three-factor, bifactor, and second-order models. [10]. Furthermore, a key component of MBI-assessed burnout, professional (in)efficacy has been shown to be a divergent factor and perhaps more suitable as an outcome of burnout than a core component [10–12].

Criticism even extends to burnout as a concept, with researchers questioning the nosological value of burnout and the accompanying prevalence estimates of the syndrome based on the grounds that no set clinical diagnostic criteria is available with different cut-off criteria being used [13]. This has led to research that posits that burnout is likely a syndrome on the depressive spectrum [14]. However, recent meta-analyses have also indicated that: i) burnout and depression are different and robust constructs [15], and ii) that the empirical relation between burnout and depression as a single point estimate may miss the more complex empirical picture [16].

Furthermore, it has been opined that burnout studies are overly concerned with the psychometric properties of instruments measuring burnout, neglecting the necessary development of theory [17]. Indeed, searching for best-fitting factor structures with statistical modelling, although informative, is mostly a data-driven exercise which often neglects theoretical considerations [18], and can also be sensitive to biases in sampling as the divergent research results do show. Nevertheless, it is important that the phenomenon under investigation is measured accurately to increase the veracity of the field – especially when it concerns employee and by extension public health. Because an instrument exists, or because it was developed first (e.g., MBI), does not mean that it necessarily captures a phenomenon more accurately than a newly developed scale. This would be a logical fallacy – especially in the context of a developing research field and advances in statistical implementations that can more accurately identify dynamics and intricacies at play in an instrument for measuring phenomena which may not have been readily available in decades past. Thus, a more useful approach in the context of the conceptualisation of burnout would be implementing a theoretical approach (scientific modelling) to model burnout to the data as opposed to purely data-driven statistical implementations. Burnout has been defined as a syndrome, that is intercorrelated components that make up a unifying concept (score). Although other burnout scales do exist, these scales either reduce and focus only on the exhaustion component (e.g., Shirom-Melamed Burnout Measure [SMBM] and the Burnout Measure [BM]) or they are based on the conceptualization and components of the MBI with some different phrasing of the items (e.g., Bergen Burnout Inventory [BBI]) [19]. All in all, there was a need for an actualised conceptualisation of burnout to investigate if all the important aspects have been covered and included.

The Burnout Assessment Tool (BAT-23) was developed using a combination of an inductive and deductive approach [19]. For the inductive approach a total of 49 practitioners were interviewed that included general practitioners, psychologists and occupational physicians who are involved at the start, middle and end of the burnout process [19]. And for the deductive approach a total of 357 items and 66 dimensions were screened before drafting the initial items of the BAT-23 – for a comprehensive description of the need for and development of the BAT-23 see the seminal article by Schaufeli et al. [19]. BAT-measured burnout is defined as: “a work-related state of exhaustion that occurs among employees, which is characterized by extreme tiredness, reduced ability to regulate cognitive and emotional processes, and mental distancing” [19]. Specifically, BAT-assessed burnout is an overall score based on four scale components. The first two components are the core components of exhaustion (feeling depleted and the inability to expend effort) and mental distance (unwillingness to expend effort, cynicism) in line also with previous research [20]. The two novel components that emerged are closely linked to exhaustion as they indicate a lack of proper management of cognition and emotions – tying into the deductive reasoning of inability and unwillingness. This was also commensurate with the inductive approach taken which included interviews with health professionals who have actively worked with burnout patients: Cognitive impairment (reduced functional capacity to adequately regulate cognitive processes) and emotional impairment (reduced functional capacity to adequately regulate emotional processes) [19, 21]. Thus, the four inter-correlated dimensions constitute one higher-order conception of burnout.

Researchers from different countries are currently conducting research with the BAT-23 and the current evidence for its validity is strong. For example, published research has shown the BAT-23 to be valid and reliable in six European countries with representative samples [22], in Japan [23] and in Korea [24]. Additionally, the BAT-23 has also been shown to be valid with Rasch analysis in both the Netherlands and Belgium (Flanders) [25]. However, no study has been conducted on the validity of the BAT-23 in Africa and the consideration of context in psychometric assessment has been shown to be important in order to offset potential biases and ensure the fair measurement and comparison between groups [26].

South Africa is a diverse multi-cultural context and the Employment Equity Act (EEA) Sect. 8 (Act 55 of 1998) requires that any psychometric instrument used in organisations, or otherwise, must be able to present evidence that it is: 1) valid and reliable, and 2) not be biased against any group or person [27]. Indeed, research has shown that levels of burnout is nuanced between gender and ethnicity and may (or may not) differ [28, 29] – indicating that equivalence of the measuring instrument is important for comparison to be accurate and fair. To this end, the construct-relevant multidimensionality of the BAT-23 is investigated with latent variable modelling to ascertain the most appropriate model in the data. Next, the convergent validity of the global burnout score of the BAT-23 is compared to that of the MBI. Then, to address the second requirement of fairness of the measurement, we also tested the measurement invariance of the BAT-23 for gender and ethnic groups.

Thus, the current study aims to investigate the validity, reliability, and measurement invariance of the Burnout Assessment Tool (BAT-23), a recently developed scale, within the South African context. To achieve these aims the following hypotheses are stated:

- H_1_: A hierarchical model of BAT-assessed burnout fits the data.
- H_2_: The BAT shows acceptable reliability coefficients.
- H_3_: There is convergent validity between the BAT and the MBI.
- H_4a_: The BAT shows invariance across gender.
- H_4b_: The BAT shows invariance across ethnicity.

## Methods

### Data collection

This study was provided ethics clearance by the Economic and Management Sciences Research Ethics Committee (EMS-REC), North-West University (Reference number: NWU-00558–17-A4). Specifically, a cross-sectional survey design was used, and participants were primarily sourced via social media (e.g., LinkedIn, Facebook) with an advertisement about the study to ensure a broader reach for voluntary participation. A national newspaper article on burnout also linked to the survey. The survey was presented in English (the accepted business language in South Africa) and included all relevant information about the purpose of the study, informed consent, voluntary participation and that the data would be handled confidentially. The sampling method was purposive as participants had to be at least 18 years of age and employed within the Republic of South Africa. The final sample consisted of 1048 employees (*n* = 1048).

### Participants

The participants comprised a purposive sample of various South African employees of at least 18 years old (*n* = 1048). The average age of the participants was 40.80 years (SD = 11.20). A slight majority of the total sample were female (*n* = 570; 54.50%) and 476 participants were male (45.50%). In terms of ethnicity, all descriptions in this section are used in line with the terminology of the Employment Equity Act, 55 of 1998 for designated and non-designated groups e.g., ‘Coloured’ is an official term in South Africa and indicates citizens of mixed ethnic origins and no offense is intended. Specifically, the sample comprised African people (36.10%), white people (37.10%), Coloured people (12.10%), Indian people (11.50%), and Asian people (3.10%). Forty-six percent of the sample had a high school education, followed by a diploma (19.10%), degree (20.00%) and post-graduate degree (14.9%). All in all, the sample was quite heterogeneous.

### Measuring instruments

The *Burnout Assessment Tool* (BAT-23) was used to measure burnout with 23 items [19, 21]. The English version of the BAT-23 was used as it is the accepted everyday business language in South Africa. Specifically, exhaustion was measured by 8 items (e.g., ‘When I get up in the morning, I lack the energy to start a new day at work’), mental distance was measured by 5 items (e.g., ‘I feel indifferent about my job’), cognitive impairment (e.g., ‘At work I struggle to think clearly’) with 5 items, and emotional impairment with 5 items (e.g., ‘At work I may overreact unintentionally’). All the BAT-23 items were measured on a 5-point rating scale ranging from Never to Always. Furthermore, the *Maslach Burnout Inventory – General Survey* (MBI-GS) was also used to measure burnout to address convergent validity [30]. Specifically, exhaustion (5 items; e.g., ‘I feel used up at the end of the workday’), cynicism (5 items; e.g., ‘I have become less enthusiastic about my work’) and professional efficacy (6 items; e.g., ‘In my opinion, I am good at my job’). All items for the MBI were measured on a 7-point rating scale ranging from Never to Always.

### Data analysis

**Fig. 1:**
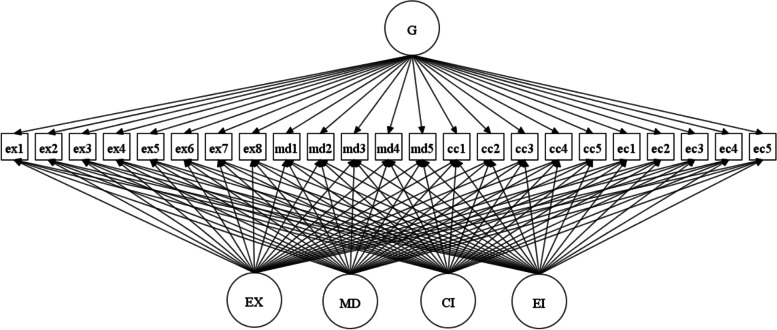
The conceptual bifactor ESEM model of the BAT-23

Latent variable modelling was implemented with Mplus 8.8 [31]. Confirmatory factor analysis (CFA) and (bifactor) exploratory structural equation modelling methods (ESEM) [(B)ESEM] were used to investigate construct-relevant multidimensionality in the data by testing different potential factor structures of the BAT with different assumptions [32, 33]. For example, the CFA model assumes that there are four correlated factors and that items do not cross-load on non-target factors – or put another way, that the cross-loadings are constrained to be exactly zero. On the other hand, ESEM does not assume exactly zero cross-loadings and blurs the lines between exploratory factor analysis and CFA by relaxing the CFA assumption to allow items to load on non-target factors with target rotation to be close to zero. Bifactor models in turn assume that there is an underlying global factor in the data with specific factors – in this case a five-factor model (a global factor and four additional specific factors), bifactor ESEM combines the ESEM and bifactor approaches with an orthogonal target rotation [33] – see Fig. 1 below.

All models were estimated with the maximum likelihood robust version (MLR) and the fit of the model(s) considered with the Satorra-Bentler chi-square, (χ^2^) comparative fit index (CFI), Tucker-Lewis index (TLI), root mean square error of approximation (RMSEA) and standardized root mean square residual (SRMR). This assisted to answer hypothesis 1. To investigate hypothesis 2, omega (composite) reliability estimates were calculated due to latent variables being used and Cronbach’s alpha not being fit for this purpose [34]. For the convergent validity (hypothesis 3), the best-fitting factor structure of the BAT-23, the BESEM model, and a BESEM model for the MBI were tested in which the global factors of the two models were correlated in a bifactor ESEM-within-CFA model implementation with syntax generated for with the help of an online tool [35]. The standard criteria for correlation effect sizes were used (small: *r* ≥ 0.10; medium* r* ≥ 0.30; *r* ≥ 0.50) in the CFA and ESEM models.

To answer the remaining hypotheses (4a, 4b) that tested for the measurement invariance between gender and ethnic groups, bifactor ESEM measurement invariance analysis was used as described by Morin [33]. Specifically in the current study, the following four types of invariances between the different groups were tested: Configural (similar factor structure – allowing for the comparison of conceptual constructs), metric (also called ‘weak invariance’; similar loading patterns – allowing for the comparison of beta coefficients in groups), strong invariance (similar intercepts – allowing for the comparison of latent means), and strict invariance (similar item uniquenesses). However, strong invariance is sufficient to meaningfully compare the latent means of groups, and strict invariance is needed when composite/total/mean score comparisons are compared [36]. To determine the level of invariance, the researchers were pragmatic in considering the thresholds for the cut-offs for delta (Δ) changes in CFI and TLI (0.010), and RMSEA (0.015) when comparing the models of increasing constraints, as the chi-square value is sensitive to a large sample size [37].

Due to a reviewer comment, supplementary analyses were also conducted with R in RStudio [38]. The ‘htmt’ function from the ‘semTools’ package was used to calculate the heterotrait-monotrait ratio (HTMT) of the correlations [39], which are equivalent to disattenuated correlations, for the four factors of the BAT-23. These values would also indicate whether the lower order factors were distinguishable from each other – HTMT values for correlations of 0.80 or below indicates discriminant validity [40].

## Results

### Construct-relevant multidimensionality of the BAT

In line with our theoretical modelling approach to this paper that burnout should be considered as a global score, Table 1 shows that the bifactor ESEM model specification of burnout was the superior fit to the data (χ^2^ = 375.55; df = 148; CFI = 0.977; RMSEA = 0.038; SRMR = 0.016; AIC = 57,638.998; SSA-BIC = 57,907.407). This evidence supported hypothesis 1.

**Table 1:** Results of the different model specifications

Model	χ^2^	d*f*	CFI	TLI	RMSEA [90%CI]	SRMR	AIC	SSA-BIC
One-factor	2695.36	230	0.746	0.720	0.101 [0.098, 0.105]	0.080	60,860.656	60,983.306
Four-factor	1011.60	224	0.919	0.908	0.058 [0.054, 0.062]	0.056	58,401.589	58,534.904
Second-order factor	1030.47	226	0.917	0.907	0.058 [0.055, 0.062]	0.058	58,423.953	58,553.714
Bifactor CFA	651.72	207	0.954	0.944	0.045 [0.041, 0.049]	0.041	57,923.074	58,086.608
ESEM	498.16	167	0.966	0.948	0.044 [0.039, 0.048]	0.022	57,796.654	58,031.290
Bifactor ESEM	375.55	148	0.977	0.960	0.038 [0.034, 0.043]	0.016	57,638.998	57,907.407

*χ2* Chi-square, *df* Degrees of freedom, *CFI* Comparative fit index, *TLI* Tucker-Lewis index, *RMSEA* Root mean square error of approximation, *SRMR* Standardised root mean square residual, *AIC* Akaike information criterion, *SSA-BIC* Sample-size adjusted Bayesian information criterion

The loadings for all model solutions are presented in Table 2. Specifically, in the bifactor ESEM model, the factor loadings (λ) for the specific factors: exhaustion ranged from 0.11 to 0.50 (*M*_*λ*_ = 0.36), mental distance from 0.15 to 0.74 (*M*_*λ*_ = 0.46), cognitive impairment from 0.38 to 0.53 (*M*_*λ*_ = 0.45), and emotional impairment from 0.41 to 0.54 (*M*_*λ*_ = 0.46). For the global factor the lowest loading on the global factor was item 2 of exhaustion (λ = 0.17; “Everything I do at work requires a great deal of effort”) which loaded higher on the exhaustion specific factor (λ = 0.48) but also cross-loaded on the mental distance specific factor (λ = 0.29) – indicating that both specific factors explain variance in this item and that this item is not a strong indicator of the global factor. In turn, the highest loading for the global factor was for item 1 of mental distance (λ = 0.78; “I struggle to find any enthusiasm for my work”), followed closely by item 5 for exhaustion (λ = 0.76; “At work, I feel physically exhausted”).

**Table 2:** Standardized Factor Loadings (λ) and Uniquenesses (δ) for the Models (*n* = 1048)

	**CFA**		**Second-order**		**Bifactor CFA**			**ESEM**					**Bifactor ESEM**					
**Item**	**λ**	**δ**	**λ**	**δ**	**G-λ**	**S-λ**	**δ**	**λ**	**λ**	**λ**	**λ**	**δ**	**G-λ**	**S-λ**	**S-λ**	**S-λ**	**S-λ**	**δ**
Exhaustion			.855	.269														
EX1	.766	.413	.767	.411	.632	.486	.364	**.824**	-.101	*-.020*	*.041*	.380	**.630**	**.495**	*-.061*	*.037*	*.029*	.399
EX2	.352	.876	.350	.878	.206	.393	.803	**.427**	.219	-.225	*-.053*	.804	**.165**	**.477**	.287	*.027*	*.042*	.798
EX3	.789	.378	.790	.375	.659	.454	.360	**.817**	-.061	-.072	.091	.364	**.661**	**.448**	*-.035*	*-.024*	*.043*	.473
EX4	.811	.342	.809	.345	.676	.486	.307	**.834**	.095	-.135	*.045*	.308	**.683**	**.466**	.090	-.075	*.005*	.378
EX5	.790	.376	.788	.379	.742	.266	.379	**.694**	.096	.086	*-.030*	.378	**.761**	**.236**	*.020*	-.077	-.108	.490
EX6	.730	.467	.728	.470	.755	*.098*	.420	**.513**	.135	.195	*-.006*	.462	**.750**	***.109***	*.037*	*-.036*	-.097	.509
EX7	.763	.418	.764	.416	.741	.200	.411	**.601**	*-.001*	.206	*.015*	.419	**.726**	**.228**	*-.039*	*.050*	*-.038*	.492
EX8	.818	.331	.820	.328	.702	.421	.330	**.859**	-.115	*.014*	*.029*	.310	**.705**	**.423**	-.096	*-.007*	*-.013*	.424
Mental distance			.782	.388														
MD1	.751	.436	.744	.447	.767	.152	.389	.385	**.291**	.226	*.002*	.431	**.775**	*.026*	**.151**	*-.041*	-.102	.467
MD2	.659	.566	.662	.561	.523	.370	.589	*.047*	**.510**	.140	*.054*	.589	**.534**	*-.042*	**.367**	*-.012*	*-.012*	.713
MD3	.597	.644	.600	.640	.322	.728	.366	-.084	**.835**	-.117	*.040*	.400	**.311**	.089	**.742**	*-.034*	.054	.559
MD4	.736	.459	.739	.453	.529	.527	.442	*.050*	**.681**	*.050*	*.034*	.440	**.537**	*-.003*	**.513**	*-.055*	*-.017*	.555
MD5	.580	.663	.585	.658	.384	.527	.574	*-.049*	**.646**	*.011*	*.066*	.572	**.362**	*.072*	**.544**	*.034*	.068	.882
Cognitive impairment			.844	.287														
CC1	.804	.353	.807	.349	.696	.377	.373	*.050*	.131	**.737**	*-.040*	.351	**.683**	*.005*	*.059*	**.420**	*-.005*	.401
CC2	.860	.260	.863	.255	.756	.391	.276	.098	.101	**.790**	*-.058*	.251	**.748**	*.000*	*.022*	**.427**	*-.028*	.247
CC3	.814	.338	.812	.341	.646	.515	.318	*-.040*	*.014*	**.771**	.094	.335	**.650**	*-.019*	*-.020*	**.493**	.102	.325
CC4	.848	.282	.846	.284	.662	.573	.234	*-.008*	*-.024*	**.849**	*.031*	.268	**.674**	*-.014*	-.061	**.531**	.059	.235
CC5	.696	.516	.692	.521	.561	.418	.510	*-.030*	-.089	**.586**	.245	.485	**.573**	*-.037*	-.102	**.377**	.187	.381
Emotional impairment			.818	.331														
EC1	.775	.400	.774	.400	.590	.502	.400	*.064*	*-.029*	*.037*	**.716**	.407	**.615**	*-.003*	*-.035*	*.052*	**.458**	.368
EC2	.822	.324	.822	.324	.631	.516	.336	*.002*	.081	*.036*	**.751**	.336	**.657**	*-.041*	.049	*.041*	**.479**	.326
EC3	.601	.639	.601	.639	.436	.423	.631	*-.013*	*.055*	*.006*	**.593**	.623	**.430**	*.071*	.074	.114	**.434**	.695
EC4	.768	.410	.769	.409	.624	.438	.418	*.045*	.084	*.054*	**.657**	.415	**.648**	*-.034*	*.046*	*.032*	**.409**	.411
EC5	.760	.422	.760	.422	.540	.565	.389	*.016*	*-.034*	*-.035*	**.809**	.388	**.560**	*.018*	*-.018*	*.058*	**.543**	.360

*CFA* Confirmatory factor analysis, *ESEM* Exploratory structural equation modelling, *G* Global factor, *S* Specific factor; target ESEM and Bifactor-ESEM factors loadings indicated in bold; non-significant parameters (*p* ≥ 0.05) indicated in italics

### Reliability and correlations

The global factor of the BAT-23 in the BESEM model indicated evidence of unidimensionality according to its omega reliability value (ω = 0.95), but the specific factors retained sufficient meaningfulness with omega values above the acceptable 0.50 threshold [41]. Specifically, the following values were observed for the specific components after considering the global factor: Exhaustion (ω = 0.68), mental distance (ω = 0.63), cognitive impairment (ω = 0.76), and emotional impairment (ω = 0.71). These results supported hypothesis 2.

As bifactor models are orthogonal, that is, the specific factors and global factor are uncorrelated, in line with the theoretical specifications of bifactor implementations, the correlations for the CFA and ESEM models are also given in Table 3 below. As can be seen from Table 3, all the factors correlated with one another with medium and large effect sizes. Specifically, the components were correlated below the cut-off of 0.85 for discriminant validity in latent variable modelling [32]. Furthermore, the size of the correlation values reduced for the ESEM (*M*_*r*_ = 0.54) compared to the CFA model (*M*_*r*_ = 0.68), supporting the notion of an underlying global factor and the decision to select the bifactor ESEM model [33].

**Table 3:** Descriptive statistics, omega reliabilities, and CFA/ESEM correlation matrix

Factors	M	SD	1	2	3	4
1. EX	2.82	0.86	(0.90/0.90)	0.53	0.66	0.61
2. MD	2.48	0.88	0.72	(0.80/0.78)	0.39	0.42
3. CI	2.08	0.83	0.71	0.63	(0.90/0.89)	0.67
4. EI	2.04	0.80	0.68	0.63	0.73	(0.86/0.85)

*CFA* Correlations below the diagonal, *ESEM* Correlations above the diagonal; omega reliability on the diagonal (CFA/ESEM), *M* Observed mean, *SD* Observed mean standard deviation, All correlations *p* < .001; *EX* Exhaustion, *MD* Mental distance, *CC* Cognitive impairment, *EC* Emotional impairment

Furthermore, the supplemental disattenuated correlations test with the HTMT method showed that the applicable correlations were all below the 0.80 cut-off for discriminant validity.

## Convergent validity of the BAT with the MBI

To determine the convergent validity of the BAT-23 with the MBI, two bifactor ESEM solutions were estimated for each measure of burnout separately. The bifactor ESEM model for the MBI had convergence issues with three specific factors, so we used the exhaustion and mental distance items in one specific factor along with professional efficacy as the second specific factor (χ^2^ = 206.39; df = 75; CFI = 0.962; TLI = 0.939; RMSEA = 0.049; SRMR = 0.028). Then, the bifactor ESEM-within-CFA solutions from each separate model were combined into one new model with only the two general burnout global factors correlated. This model was a good fit to the data (χ^2^ = 1579.39; df = 603; CFI = 0.937; TLI = 0.922; RMSEA = 0.039; SRMR = 0.045). The results showed that the BAT-23’s global factor was highly correlated with the MBI’s global factor (*r* = 0.904; large effect); a large overlap in variance (81.72%), indicating convergent validity of the instruments and support for hypothesis 3.

Furthermore, the BAT-23 showed a clearer global factor and specific factors than the MBI did due to the low negative loadings of professional efficacy on the MBI global factor (-0.432 to -0.080; *M*_*λ*_ = 0.255) and the stronger positive loadings of the professional efficacy specific factor (0.432 to 0.672; *M*_*λ*_ = 0.525). This evidence supported (as in previous studies on the factor structure of the MBI) that the professional efficacy component was mostly a divergent factor from the global MBI factor that should be considered independently from the burnout core.

### Measurement invariance

Concerning the measurement invariance of the second-order model of the BAT-23. Hypotheses 4a and 4b were accepted as stated – see Table 4 below for the results of all the invariance tests. The models gender (configural, metric, strong, and strict) did not violate the cut-off criteria set for the delta changes to CFI, TLI, and RMSEA [37] – strict invariance was therefore supported. Female employees scored higher than male employees on global burnout (*d* = 0.36; small effect), but male employee scored higher on the specific factor of mental distance (*d* = 0.37; small effect). However, hypothesis 4b for invariance based on ethnic group was only supported for strong invariance which indicated the possibility of comparison of latent mean levels between the groups, but strict invariance was lacking. However, an investigation of partial strict measurement invariance indicated that if the uniquenesses of seven items were freed (EX1, EX4, EX5, MD2, CC1, CC2, EC3), then partial strict invariance was achieved. Note that the ethics clearance for this study did not allow for direct comparison of means for ethnic groups, only whether the BAT-23 is an equivalent measure. Therefore, no mean comparison was performed.

**Table 4:** Results of the BESEM measurement invariance testing for gender and ethnicity

**Model: Gender**	***χ***^**2**^	**d*f***	**CFI**	**TLI**	**RMSEA**	**CM**	**ΔCFI**	**ΔTLI**	**ΔRMSEA**
M1: Configural	590.40	296	.970	.949	.044 [.038, .049]	-			
M2: Metric (λ)	672.22	386	.971	.962	.038 [.033, .042]	M1	+ .001	+ .013	-.006
M3: Strong (λ, ν)	709.59	404	.969	.962	.038 [.033, .043]	M2	-.002	.000	.000
M4: Strict (λ, ν, δ)	775.86	427	.965	.958	.040 [.035, .044]	M3	-.004	-.004	+ .002
**Model: Ethnicity**	***χ***^**2**^	**d*f***	**CFI**	**TLI**	**RMSEA**	**CM**	**ΔCFI**	**ΔTLI**	**ΔRMSEA**
M1: Configural	984.92	592	.964	.939	.051 [.046, .057]	-			
M2: Metric (λ)	1319.37	862	.958	.951	.046 [.041, .051]	M1	-.006	+ .012	-.005
M3: Strong (λ, ν)	1460.37	916	.950	.945	.049 [.044, .053]	M2	-.008	-.006	+ .003
M4: Strict (λ, ν, δ)	1968.50	985	.911	.908	.063 [.059. .067]	M3	-.039	-.037	+ .014
M5: Partial Strict	1629.35	977	.941	.939	.052 [.047, .056]	M3	-.009	-.006	+ .003

*χ*^*2*^ Robust chi-square, *df* Degrees of freedom, *CFI* Comparative fit index, *TLI* Tucker-Lewis index, *RMSEA* Root mean square error of approximation with 90% confidence interval of the RMSEA, *λ* factor loadings, *ν* intercept, *δ* uniquenesses, *CM* Comparison model, *ΔCFI* Change in CFI, *ΔTLI* Change in TLI, *ΔRMSEA* Change in RMSEA

## Discussion

The aim of this study was to investigate the validity, reliability, and measurement invariance of the BAT-23 – a new burnout instrument. Results from the latent variable modelling supported hypothesis 1 as the bifactor ESEM model of burnout was the most appropriate model. This result also supports the recent research that found support for the validity of the BAT-23 and that burnout assessed by the BAT-23 can be considered as an overall global score [19, 22, 23, 25]. The omega reliability coefficients were all acceptable for the CFA, ESEM, and bifactor ESEM solutions – supporting hypothesis 2. That is, an overall global burnout score indicated by the items of the instrument and four meaningful specific factors may also be interpreted. Therefore, there is some evidence for multidimensionality and strong evidence for a unidimensional interpretation, supporting our overall expectations.

Furthermore, hypothesis 3 was also supported as the BAT-23 and MBI showed convergent validity, specifically a shared variance indicating that a similar phenomenon is being measured by the two scales. Some might then question why a new measure for burnout is needed at all then? However, the BAT-23 answers many of the criticisms levelled at the MBI and other measures [19], as it provides an updated conceptualisation of burnout and produces a single burnout score. Specifically, the content of the measurement of burnout was also updated with the addition of the cognitive impairment and emotional impairment components, equally relevant for intervention considerations. This helps to advance the field of potential intervention on employee burnout. Consequently, all four components are necessary for considering an employee’s global burnout score and they should not be used in isolation to consider burnout risk.

In terms of the measurement equivalence of the BAT-23, it showed at least strong measurement invariance for gender and ethnicity. Specifically, gender not only showed strong measurement invariance, but also strict measurement invariance (supporting hypothesis 4a). Female employees scored higher on global burnout than male workers with a small effect size and males scored higher on the mental distance specific factor, both these results are in line with review studies on burnout level in these groups [28, 29]. Lastly, invariance was also tested based on ethnicity. Here, only strong invariance was found but not strict invariance. However, partial strict measurement invariance was found in a subsequent analysis (support for hypothesis 4b). This result indicates that latent means scores can be tested for the groups if required. Due to ethics clearance restrictions, this study could not compare ethnic groups directly. However, the strong and partial strict measurement invariance evidence shows that the BAT-23 measures equivalently as a measure and does present the utility to compare scores across groups, if required. As this is the first study to investigate the BAT-23 in the African context there is no previous literature to compare the invariance based on ethnic groups, but previous research has shown that the MBI presented evidence for equivalence in different ethnic groups of police and emergency medical technician samples in South Africa [42, 43].

Even though there may have been a recent imbalance of studies concerning the psychometric properties of instruments in burnout research [17], it remains critical for researchers to consider any legal and ethical obligations and their theoretical assumptions when it comes to the operationalisation of burnout for the advancement of theory. That is, if burnout is considered a syndrome, as per its definition, it should be operationalised in statistical models in a similar way and the research results show that the BAT-23 can successfully operationalise burnout as a hierarchical solution with the needed validity and reliability. Therefore, in practice, the operationalisation of burnout as syndrome is an important consideration as this implies that a global burnout score must be considered for an employee potentially suffering burnout and not just certain scores. All in all, researchers should carefully consider their operational definition of burnout, the associated measures, and statistical model that will be valid in future research studies.

## Limitations and recommendations for future research

This study used purposive sampling, a non-probability method to collect the data. However, the sample was relatively large and heterogeneous, but admittedly not completely representative of the South African population. However, it is important to note that fully representative samples are quite rare within the South African context. Consequently, generalising the results should be done with caution. Furthermore, a cross-sectional design was used and therefore predictive validity of the BAT-23 over time should still be established with longitudinal data. Additionally, different language versions of the BAT-23 could also be created and validated in South Africa (e.g., Afrikaans, Setswana, isiZulu). Furthermore, even though the seminal article on the BAT-23 showed discriminant validity from depression as measured by a subscale of the 4DSQ [44], it would have been ideal if the current study also included a measure of depression to investigate with the BAT-23 in this context. Finally, this study approached the modelling of burnout in line with its definition as a syndrome with a global measurement. However, to fully support this claim, time intensive studies are necessary that need to consider the development of symptoms that can lead to clinical diagnostic criteria.

## Conclusion

Burnout continues its upward trajectory as a public health concern, which will be compounded in the current and post-pandemic world. The Burnout Assessment Tool (BAT-23) is a valid and reliable measurement for the risk of burnout as a global score within the South African context. Additionally, the results revealed the tendency of professional efficacy as a divergent factor from the core burnout syndrome in the MBI. All in all, there is now a contemporary, updated conceptualisation of burnout and a another psychometrically sound scale to measure it within the Southern African context.

## Data Availability

The datasets used and/or analysed during the current study can be requested from the corresponding author who will consider all reasonable requests.
